# Complete genome sequence of *Bacillus subtilis* phage fHSPT3

**DOI:** 10.1128/mra.00404-24

**Published:** 2024-07-31

**Authors:** Tushar Midha, Aaina Choudhary, Somesh Baranwal

**Affiliations:** 1 Department of Microbiology, Central University of Punjab, VPO Ghudda, Bathinda, Punjab, India; Department of Biology, Queens College, Queens, New York, USA

**Keywords:** *Bacillus*, phage

## Abstract

Here, we describe a *Bacillus subtilis* bacteriophage isolated from sewage water. The phage fHSPT3 was isolated against the host *Bacillus subtilis* 168 and has a genome size of 150,187 bp with 221 protein-coding sequences. The fHSPT3 belongs to the genus *Siophivirus*, lacks virulence or antibiotic resistance genes, and shows a virulent life cycle predicted through PhageScope and PhageAI.

## ANNOUNCEMENT

*Bacillus subtilis* is a Gram-positive, rod-shaped, spore-forming model organism with applications in food fermentation, enzyme production, feed additive, and plant biocontrol (1, 2). We report a complete genome of *Bacillus subtilis* phage fHSPT3 belonging to the genus *Siophivirus* isolated from influent sewage water located at Dabwali, Haryana, India (N29°56′18.56″, E74°41′5.497″).

**Fig 1:**
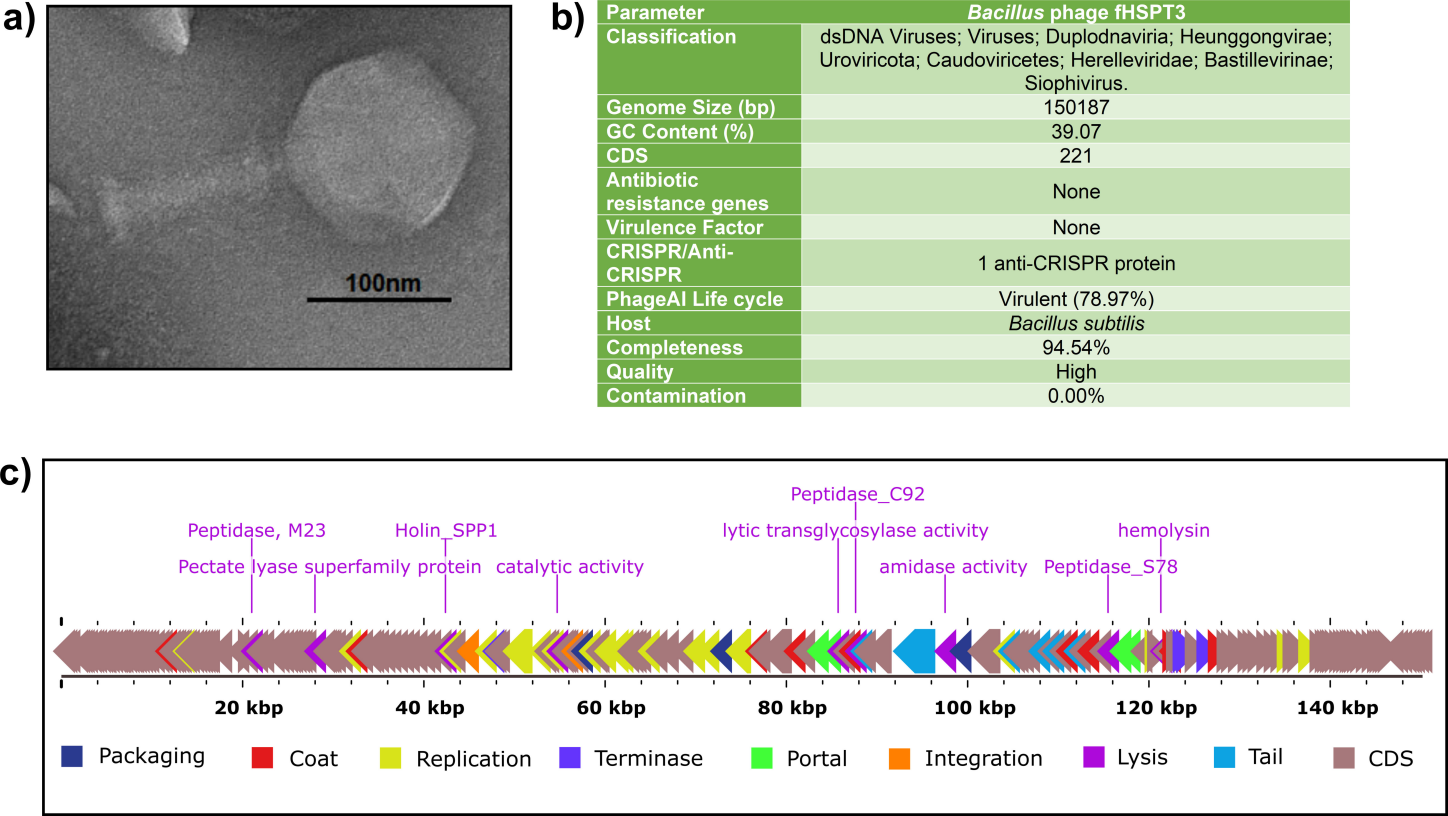
Details of *Bacillus subtilis* phage fHSPT3. (a) Morphology of phage observed through transmission electron microscopy. (b) Genomic details, and (c) genomic map of phage fHSPT3 depicting various protein-coding regions.

The phage was isolated after enrichment with host *Bacillus subtilis* 168 (BEI resources, NIAID, NIH) and purified with three rounds of double-layer agar overlay method to get monophage (3). Briefly, 2 mL water sample was filtered with a 0.22 µm filter (HiMedia) and incubated at 37°C with 200 µL overnight host bacterial culture in 3 mL double strength tryptic soy broth for 6 h with continuous shaking at 120 rpm. Then, it was centrifuged at 8,000 *g* for 5 minutes; 500 µL supernatant was used for a double-layer plate approach, mixed with 500 µL of log-phase *Bacillus subtilis* 168 and 5 mL of molten top agar, and plated on tryptic soy agar (HiMedia) plate and left to incubate overnight at 37°C. A plaque with clear, brilliant, and smooth edges was inoculated into 500 µL of SM buffer pH 7.5. For transmission electron microscopy, fHCPT3 phage lysate was stained with 2% phosphotungstic acid (pH 7.0) for 15 seconds after treatment with ammonium acetate (4). The stained grid was observed with Tecnai G20 HR-TEM at SAIF, AIIMS, New Delhi. The length of the phage was determined to be 275 ± 01 nm with a head width of 133 ± 2 nm using Image J 1.54 software (NIH, Bethesda, MD, USA) (Fig. 1a). Phenol-chloroform-based extraction was used for DNA isolation from phage lysate following treatment with DNase to remove the host DNA contamination (3).

The purified phage DNA was outsourced to the National Institute for Biomedical Genomics, Kalyani (India) for library preparation through NexteraXT (Illumina) and Illumina pair-based whole genome sequencing through NovoSeq 6000-2 × 250 bp SP v1.5-150x (Illumina Inc.). FastQC 0.11.7 was performed to check the quality. A *de novo* genome assembly was performed with Q.C. pass reads through Unicycler version 0.4.8, functioning as SPAdes-optimiser 3.13.0 (5). PhageTerm 1.0.12 and QUAST 5.2.0 were used to determine phage termini and assembly quality. A single contig with 94% mapping reads was obtained for phage fHSPT3 (6, 7). Then, the genome was annotated with Prokka 1.14.6 (8) and further with PhageScope to determine host and completeness (9). Phagescope uses CheckV version 1.0.1 to determine contamination and completeness. The phage fHSPT3 genome is a complete linear genome of 150,187 bp with 39.07% GC content and is of high quality. A total of 221 protein-coding sequences were obtained without any tRNA, antibiotic-resistant gene, virulence factor, or CRISPR elements. The phage life cycle was determined to be virulent with 78.97% confidence with PhageAI 1.0.2 tool (10) (Fig. 1b). A genomic map was prepared depicting the lysis proteins with Proksee version 1.0.0a6 (Fig. 1c) (11). The NCBI BLASTn (12) showed that phage fHSPT3 is closely related to genus *Siophivirus* and family Herelleviridae phages, *Bacillus* phage vB_BspH_Mawwa (MW749002.1) and *Bacillus* phage vB_BspH_TimeGriffin (MW749007.1) with a percentage identity of 98.03% and 97.75%, respectively, and query cover of 92% and 90%, respectively.

## Data Availability

The assembly of the phage fHSPT3 genome is deposited in GenBank with accession no. PP626411 and associated raw data were deposited under SRA accession no. SRR28698409, BioProject accession no. PRJNA1098433, and BioSample accession no. SAMN40906862.
